# Open reduction and internal fixation with bone grafts for comminuted mason type II radial head fractures

**DOI:** 10.1186/s12891-018-2214-4

**Published:** 2018-08-16

**Authors:** Guanyi Liu, Erman Chen, Dingli Xu, Weihu Ma, Leijie Zhou, Jianming Chen, Zhijun Pan

**Affiliations:** 1 Department of Orthopedics, Ningbo NO.6 Hospital, 1059 Zhongsandong Road, Ningbo, Zhejiang, 315040 People’s Republic of China; 2 Department of Orthopedics, the Second Affiliated Hospital, School of Medicine, Zhejiang University 88 Jiefang Road, Hangzhou Zhejiang, 310009 People’s Republic of China

**Keywords:** Elbow, Radial head fracture, Internal fixation

## Abstract

**Background:**

The use of bone graft for the radial head fractures has been previously described and occasionally used by other authors.This is the first paper, to my knowledge, dealing with the relevant issue about the importance that the use of an autologous bone graft can have on the radial head fractures.

**Methods:**

From July 2010 to July 2014, 20 consecutive patients who underwent open reduction and internal fixation for a closed Mason type II radial head fracture were retrospectively reviewed. Patients with Mason type I, III, simple type II, and comminuted type II fractures treated without bone grafting were excluded. A clinical examination and radiographic evaluation were performed. The overall functional result was evaluated using the Mayo Elbow Performance Score (MEPS). The Broberg and Morrey classification was used to evaluate traumatic arthritis.

**Results:**

The average follow-up duration was 31 months (range, 24–50 months). Bone union of the radial head fracture was achieved in all patients at an average of 13.5 weeks (range, 12–17 weeks). Postoperative radiographs showed no cases of postsurgical ligamentous instability, necrosis of the radial head, or internal fixation failure. The mean range of motion of the affected elbow was 128° ± 8.4° in flexion, 14.5° ± 11.1° in extension, 68.7° ± 14.1° in pronation, and 65.2° ± 18.2° in supination. The mean MEPS was 92 ± 7.9 points (range, 80–100); the outcome was excellent (90–100 points) in 13 patients and good (75–89 points) in 7 patients. The MEPS tended to be higher in patients with an isolated fracture (*p* = 0.016). Based on the Broberg and Morrey classification for radiographic assessment of post-traumatic arthritis, 15 elbows had no evidence of degenerative changes (grade 0), and 5 elbows had grade 1 changes.

**Conclusion:**

Although radial head fractures may not be amenable to internal fixation, our findings suggest that open reduction and internal fixation with an autogenous bone graft from the lateral epicondyle of the humerus provides satisfactory elbow function in patients with comminuted Mason type II radial head fractures.

## Background

**Fig. 1:**
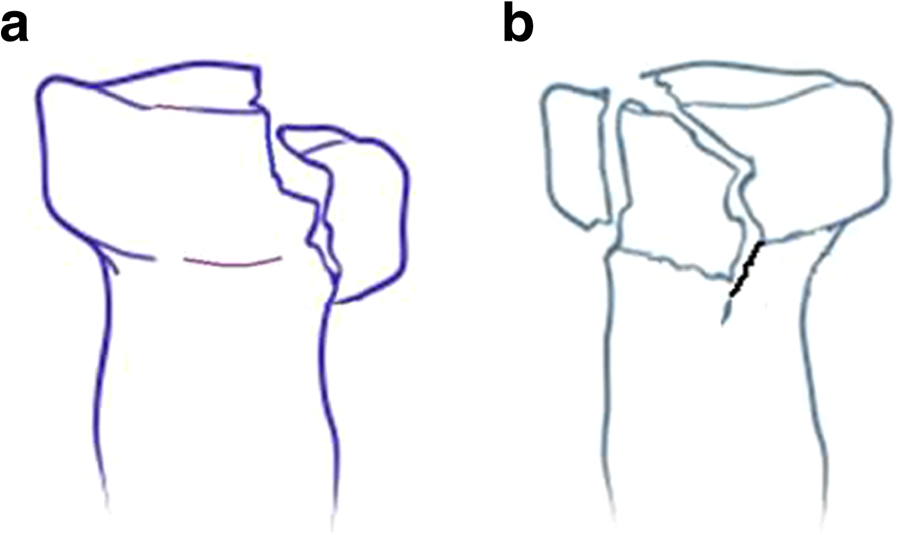
Mason type II fractures of radial head include simple (**a**) and comminuted (**b**) partial articular fractures with displacement

Radial head fractures are common injuries and usually classified according to the original Mason classification: type I, fissure fractures or marginal sector fractures without displacement; type II, partial articular fractures with displacement; and type III, comminuted fractures involving the whole head of the radius [1]. Type II actually includes simple and comminuted partial articular fractures (Fig. 1). The operative treatment of comminuted type II radial head fractures has been controversial because of conflicting evidence supporting resection, prosthetic replacement, and open reduction and internal fixation (ORIF) [1–8]. Improvements in operation techniques and implants such as low-profile mini-plates have increased the appeal of internal fixation treatment, especially for comminuted type II fractures of the radial head [5–7]. To our knowledge, ORIF with an autogenous bone graft from the lateral epicondyle of the humerus for comminuted Mason type II radial head fractures has rarely been reported. This technique was used to treat 20 patients in our study and produced desirable outcomes.

## Methods

From July 2010 to July 2014, 40 consecutive patients who underwent ORIF of a closed Mason type II radial head fracture (simple fractures, *n* = 11; comminuted fractures, *n* = 28) performed by the senior author (G.Y.L.) at our institution were identified and retrospectively case studied. Of the 28 patients with comminuted fractures, 22 were treated by ORIF with an autogenous bone graft and 6 without. Patients with Mason type I, III, simple type II, and comminuted type II fractures treated without bone grafting were excluded.

The study protocol was approved by our institution’s research ethics board. After reviewing the patients’ medical records and radiographs in our electronic medical record and image databases, we invited the patients to our hospital for clinical and radiographic evaluations. Written informed consent was required for participation in this study. Two patients were lost to follow-up. Thus, the final study population comprised 20 patients.The patients comprised 12 men and 8 women with a mean age of 47 years (range, 20–65 years). They had 14 dominant injuries and 6 nondominant injuries. The mechanism of injury was a fall from a standing height in three patients, a fall from a greater height (> 3 m) in six patients, a traffic accident in eight patients, and a sports injury in three patients.

All patients had a comminuted Mason type II radial head fracture (a fracture involving the partial articular surface, splitting it into two or more fragments). According to the comprehensive classification of fractures, the patients had 3 type B fractures (all were B2.3) and 17 type C fractures (10 were C2.3 and 7 were C3.2). Three radial head fractures were isolated injuries; 17 were associated with a fracture-dislocation of the elbow joint (Mason–Johnston type IV) [1, 2]. Twelve patients had elbow terrible triad injuries, two had transolecranon fracture-dislocation of the elbow, two had Bado type II Monteggia fractures, and one had a radial head fracture combined with dislocation of the elbow [9]. The Bado type II fractures were subclassified based on the location of the ulnar fracture according to the Jupiter classification [10]. Both fractures were at the level of the coronoid process (type A). The coronoid fractures in patients with elbow terrible triad injuries were classified according to Regan-Morrey [11]. Three fractures were associated with minor coronoid avulsion fractures (Morrey type I), eight were associated with a Morrey type II fracture, and one was associated with a Morrey type III fracture.

### Operative technique

The operative details varied depending on the indication for surgery and the numbers of fractures and injured ligaments. A brachial plexus block was used in all patients. Patients with an elbow terrible triad injury were placed in the supine position with the involved arm draped on the hand table. Patients with Monteggia fractures and transolecranon fracture-dislocation of the elbow were positioned in the lateral decubitus position with the limb supported over a bolster. Under pneumatic tourniquet control, the fracture was approached through an extended lateral approach for patients with an elbow terrible triad injury and through a midline longitudinal dorsal incision for those with Monteggia fractures and transolecranon fracture-dislocation of the elbow.

The radial head and lateral collateral ligament complex were exposed and checked. A longitudinal incision was made through the annular ligament and capsule, along the lateral collateral ligament. The radial head fracture was identified, and all pieces of the radial head were found following irrigation of the intra-articular hematoma. Care was taken to protect the synovium and periosteum overlying the fragments to preserve the vascular supply and facilitate reduction [12]. Once reduced, the fracture fragments were temporarily fixed with 1-mm Kirschner wires.

In this series, autogenous bone grafts from the lateral epicondyle were implanted in the bone defect of the radial head fractures. The donor site was located in the lateral metaphysis of the humerus and far away from the origins of the lateral collateral ligamentous complex and common extensor tendon. The size of the bone graft was commensurate with the injury. Both cancellous and corticocancellous bone were taken. Bone chips were implanted in the bone defect to maintain the reduction of fractures and/or buttress fragments of the articular surface.

Definitive fixation was then secured using countersunk head screws, Kirschner wires, mini locking plates, or combinations of these methods. The radial head fracture was fixed with Kirschner wires alone in one patient, with a low-profile mini-plate alone in six, with countersunk head screws alone in four, and with a combination of a plate and countersunk head screws in nine.

We used a low-profile T- or Y-shaped mini locking plate with a 0.55-mm profile height (F3 fragment plating system; Johnson & Johnson Co., New Brunswick, NJ, USA). The screws were 2.0 mm in diameter and could be countersunk into the plate. The plates were placed on the nonarticulating portion (“safe zone”) of the radial head [13, 14]. The nonarticulating portion of the radial head exhibited a thinner band of yellowish cartilage relative to the wider, white, glistening cartilage of the articular portion of the radial head [15]. However, placement of mini-plates was not limited to the nonarticular portion of the radial head because they could be applied as a buttress from the radial shaft to the head or to the lower corner of the radial head [5]. Sometimes a fragment was too small to be fixed with a plate or screw and could only be fixed with 1-mm Kirschner wires (two patients).

Associated fractures of the coronoid process and olecranon were fixed concomitantly. After fixation, the stability of the fixation and the range of movement of forearm rotation were checked. The radial head and proximal radioulnar joint were examined by fluoroscopy to ensure extra-articular screw placement. The capsule and annular ligament were closed with absorbable sutures. Avulsion of the lateral collateral ligament occurred in 13 patients; reattachment to the lateral condyle was performed with a transosseous suture in 4 patients and with bone anchors (Johnson & Johnson Co., America) in 9 patients [16, 17]. Finally, stability of the elbow was tested by the hanging arm test in patients with terrible triad injuries [18]. If unacceptable instability persisted, then the medial collateral ligament was exposed and repaired (in two patients).

### Postoperative management

Functional aftercare without immobilization was carried out in patients with isolated radial head fractures; otherwise, postoperative immobilization in a splint or cast was performed for an average of 2 weeks (range, 3 days to 3 weeks) followed by active exercises as tolerated by the patients. Partial weight bearing began at the sixth postoperative week. Full weight bearing was delayed until the fracture was healed and a callus appeared on radiographs.

### Evaluation

The clinical examination and radiographic evaluation were performed by an independent observer. Flexion and extension of the elbow and wrist (forearm in neutral rotation) and the range of pronation and supination were measured. The overall functional result was evaluated using the Mayo Elbow Performance Score (MEPS) [19]. Follow-up radiographs, including anteroposterior and lateral projections of the elbow, were used to assess fracture union, congruity, post-traumatic osteoarthritis, and hardware-related problems. In patients with equivocal X-rays, reconstructed computed tomography scans were obtained for further assessment. The Broberg and Morrey classification was used to evaluate traumatic arthritis [19].

### Statistical analysis

Data analyses were performed with SPSS 18.0 (SPSS Inc., Chicago, IL). Continuous variables are presented as mean ± standard deviation. A two-sample t test was performed to compare the ROM and MEPS between patients with an isolated fracture of the radial head and those with a more complex injury pattern. All *p* values were two-sided, and values < 0.05 were considered statistically significant.

## Results

### Result of the entire elbow injury

The clinical results are summarized in Table 1. All patients underwent operative treatment without neurovascular complications. The average follow-up duration was 31 months (range, 24–50 months). Postoperative radiographs showed no cases of postsurgical ligamentous instability and internal fixation failure. We found no serious persistent pain of the elbow, nerve impairment, complex regional pain syndrome, or infection.

**Table 1:** Characteristics of patients in the present series

Case	Age	Gender	Diagnosis	Follow-up(months)	Extension(°)	Flexion(°)	Pronation(°)	Supination(°)	MEPS
1	40	Male	radial head fracture	28	0	140	70	75	95
2	47	Male	radial head fracture	50	5	130	80	85	100
3	63	Male	radial head fracture	40	10	125	80	75	100
4	29	Female	radial head fracture and dislocation	35	10	120	80	65	95
5	62	Female	terrible triad injury	25	25	110	35	60	80
6	59	Male	terrible triad injury	29	35	125	60	40	85
7	49	Male	Monteggia fracture	30	25	125	75	55	95
8	51	Female	Monteggia fracture	26	15	135	75	70	95
9	65	Male	terrible triad injury	27	25	130	75	85	100
10	58	Male	terrible triad injury	24	25	120	60	30	80
11	43	Female	terrible triad injury	40	15	130	85	80	100
12	41	Female	terrible triad injury	39	5	135	55	45	85
13	46	Male	terrible triad injury	32	0	120	70	70	85
14	47	Female	terrible triad injury	27	5	125	65	50	85
15	29	Female	terrible triad injury	26	20	130	65	60	95
16	55	Male	transolecranon fracture-dislocation	24	15	135	70	75	100
17	65	Male	terrible triad injury	26	10	125	70	70	90
18	20	Male	terrible triad injury	40	25	135	60	55	95
19	28	Male	transolecranon fracture-dislocation	30	15	135	75	85	95
20	43	Female	terrible triad injury	28	5	130	70	75	85

The mean ROM of the affected elbow was 128° ± 8.4° in flexion, 14.5° ± 11.1° in extension, 68.7° ± 14.1° in pronation, and 65.2° ± 18.2° in supination with a flexion–extension arc of 113° ± 16.5° and pronosupination arc of 134° ± 28°. The mean MEPS was 92 ± 7.9 points (range, 80–100 points); the outcome was excellent (90–100 points) in 13 patients and good (75–89 points) in 7 patients. The MEPS tended to be higher in patients with an isolated fracture (*t* = 3.155, *p* = 0.016). Based on the Broberg and Morrey classification for the radiographic assessment of post-traumatic arthritis, 15 elbows had no evidence of degenerative changes (grade 0), and 5 elbows had grade 1 changes. There was no incidence of grade 2 or 3 changes at the final follow-up. Three of the 20 patients (15%) had evidence of periarticular ossification. In all three patients, the periarticular ossification was minimal.

### Result of fractures of the radial head

**Fig. 2:**
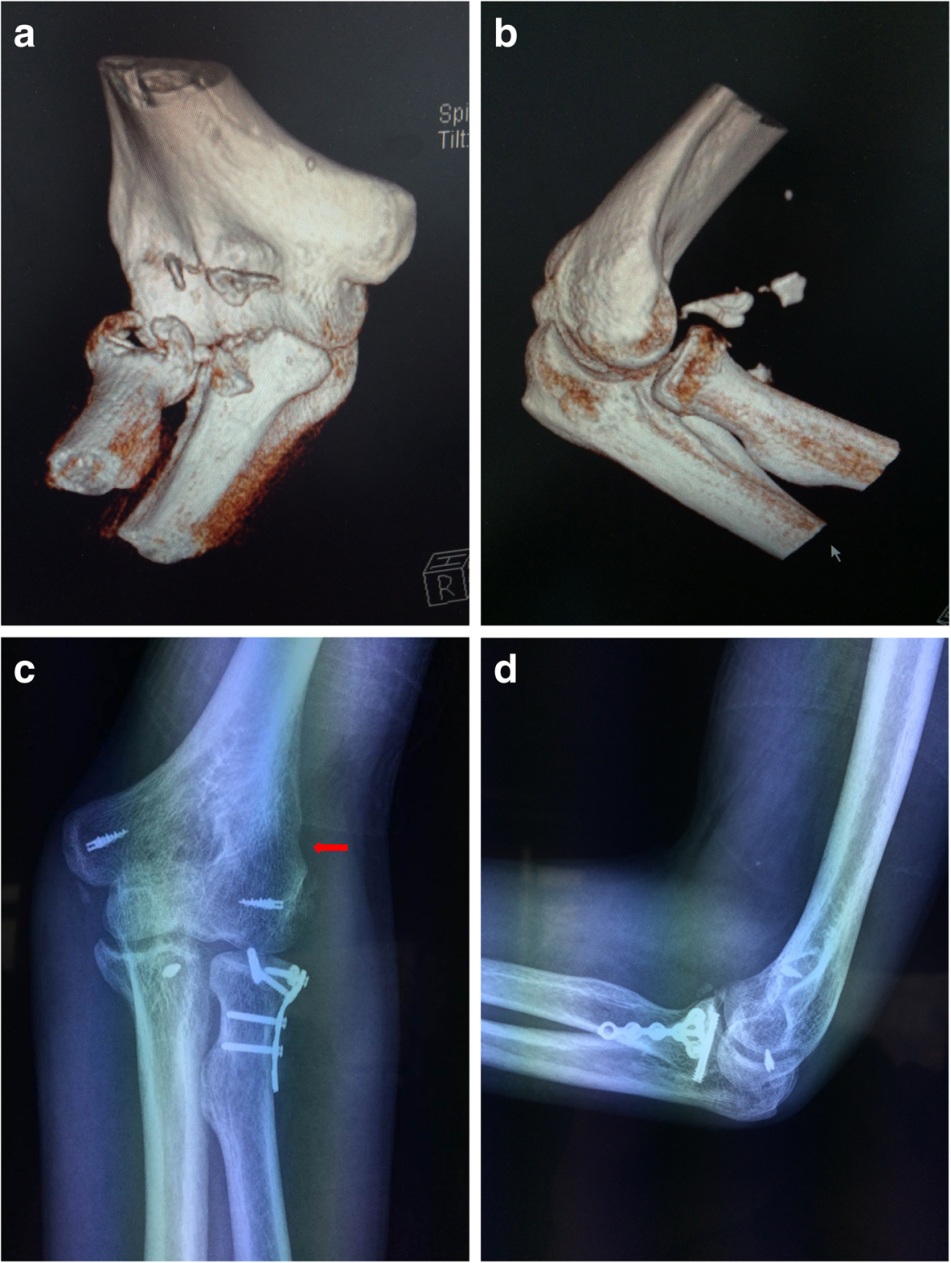
A typical case. A 59-year-old man sustained a terrible triad elbow injury and was treated though a lateral approach combined with a medial approach (Case 9). Open reduction and internal fixation with a bone graft for the radial head fracture and repair of the lateral collateral ligament as well as fixation of the coronoid fracture and repair of the medial collateral ligament were performed. **a**, **b** Preoperative CT scans showed a comminuted Mason type II radial head fracture and a coronoid fracture. **c**, **d** Postoperative radiographs at the last follow-up demonstrated good bone union. The donor site could be seen on the anteroposterior radiograph of the elbow (red arrow)

Bone union of the radial head fractures was achieved in all patients at an average of 13.5 weeks (range, 12–17 weeks) (Fig. 2). No complications occurred in the donor site area. Postoperative radiographs showed no cases of necrosis of the radial head, reduction loss, or internal fixation failure. No serious persistent pain and degenerative change of the lateral side of the elbow was found. None of these patients had a severe restriction of pronosupination or flexion–extension. Implant removal was performed in one patient.

## Discussion

To our knowledge, this is the largest series of radial head fractures treated by ORIF with autogenous bone grafts. The main findings of the present study are as follows. (1) ORIF with an autogenous bone graft from the lateral epicondyle is an effective treatment for comminuted Mason type II radial head fractures. (2) Patients with an isolated fracture of the radial head tended to have better elbow function results. (3) Comminuted partial articular fractures of the radial head were more likely to be part of a serious complex injury pattern than to be isolated fractures.

The aim of the treatment of radial head fractures is to restore good elbow function. Radial head excision has been advised for comminuted fractures [3, 6]. However, excision of the radial head may be associated with proximal radial shaft migration and consequent disorders in the wrist [3, 6]. Radial head fractures combined with injury of the medial ligament of the elbow should not be treated by radial head excision [20–22]. Prosthetic replacement of the radial head is another alternative and is commonly performed when the head is fractured into more than three displaced fragments. However, complications associated with prosthetic replacement have been reported [25]. Flinkkilä et al. [23] reported that loosening of press-fit radial head prostheses was found in 12 of 37 patients (32.4%). van Riet et al. [24] reported that 47 elbows underwent removal of a failed metallic radial head replacement.

ORIF is the preferred treatment method for radial head fractures, particularly in young and active patients. Both biomechanical and clinical studies have proven that preservation of the radial head has a positive impact on elbow stability [25–27].

ORIF is good choice for treating type II fractures with a single fragment. However, Mason [1] recommended resection for type II partial articular fractures with more than one fragment because the risk of radial head necrosis and secondary loss of reduction seems high in comminuted radial head fractures. Heim et al. [28] encountered problems with healing or avascular necrosis in 5 of 11 comminuted fractures. Autogenous bone grafts are beneficial for both maintenance of reduction and healing of fractures. In the present series, no nonunion or avascular necrosis occurred after ORIF with a bone graft from the lateral epicondyle.

However, bone grafting has not been widely used in combination with ORIF for treatment of radial head fractures, and different donor sites have been reported in the literature [7, 29–31]. Ring et al. [7] applied ORIF with an autogenous bone graft to 1 of 30 patients with Mason type II fractures. Oztürk et al. [29] reported that 15 patients with radial head fractures underwent ORIF, and bone grafts from the distal part of the radius were used in 5 patients. Patel et al. [30] treated a severe comminuted fracture of the radial neck by ORIF with a tricortical iliac crest bone graft. Luenam et al. [30] reported that union was achieved in 9 of 10 patients who underwent an operation using an iliac crest bone graft for partial radial head reconstruction in the treatment of complex dislocation of the elbow. We prefer autografting from the lateral epicondyle of the humerus. The reasons for this choice are as follows: (1) taking bone chips from the same incision is convenient and induces less iatrogenic damage; (2) preparation of another sterile area for donation is not needed; and (3) the procedure is safe and has no adverse effects on elbow function.

Union of radial head fractures and good or excellent elbow function were achieved in all patients. The mean MEPS in this series was 92 points with a flexion–extension arc of 113° and pronosupination arc of 134°. The outcomes in our cohort were comparable with or better than other results reported in previous studies with similar patient populations. Ring et al. [7] reported a mean functional index of 92 points in 30 patients with Mason type II fractures using ORIF and a mean range of movement of 11° to 130° at the elbow (mean of 72° of pronation and 72° of supination). They found a trend toward unsatisfactory results with severe restriction of forearm rotation in patients with comminuted Mason type II fractures and unsatisfactory results in four patients. In our series, however, only two patients had forearm rotation of < 100° and none had fair or poor functional results. Both patients with restricted forearm rotation had a terrible triad injury of the elbow. Ring et al. [7] also found that patients with radial head fractures exhibiting a complex elbow dislocation pattern were more likely to have an unsatisfactory treatment result. We obtained the same finding in our series; patients with an isolated radial head fracture had a much higher MEPS than patients with a fracture and dislocation of the elbow.

Comminuted partial articular fractures of the radial head were more likely to be part of a complex elbow dislocation pattern. We noted that most such fractures (17 of 20 patients) were associated with fracture and dislocation of the elbow in this series. Pugh et al. [17] recommended prosthetic replacement for a radial head fracture with comminution or impaction in terrible triad injuries. Ring et al. [32] resected the radial head in 4 of 11 patients with terrible triad injuries, and 7 patients reported unsatisfactory results. All four patients with a satisfactory result were treated by ORIF for the radial head fracture. Ring et al. [7] suggested that when ORIF is impossible, resection of the radial head and prosthetic replacement should be considered for complex injury patterns. However, we consider that even when two or more fracture fragments are present, the primary goal is to fix the type II partial articular fracture. The radial head is important to elbow stability in this situation and therefore should not be resected. Prosthetic replacement should be performed when a radial head fracture is irreparable in complex injury patterns [17].

There are some limitations of this study. This is a retrospective study and has the biases of this study design with a small sample size. Future research with a randomized controlled trial may help to draw a conclusion of how much the bone graft can attribute to the obtained results. The accurate indications for ORIF with bone grafts for these fractures also need to be explored.

## Conclusion

Although radial head fractures may not be amenable to internal fixation, our findings suggest that ORIF with an autogenous bone graft provides satisfactory elbow function in patients with comminuted Mason type II radial head fractures.

## Abbreviations

MEPS: Mayo Elbow Performance Score
ORIF: open reduction and internal fixation
ROM: range of motion
